# Concurrent hereditary angioedema type I and common variable immunodeficiency presenting as suspected antibiotic hypersensitivity: Case report and literature discussion^☆^

**DOI:** 10.1016/j.waojou.2026.101407

**Published:** 2026-05-22

**Authors:** Delia Urdea, Deniss V. Mereuta, Raluca E. Iorga, Mariana Pavel-Tanasa

**Affiliations:** a Grigore T. Popa University of Medicine and Pharmacy Iasi, 700115 Iasi, Romania; b Laboratory of Immunology, St. Spiridon Emergency Clinical County Hospital, 700106 Iasi, Romania

**Keywords:** Hereditary angioedema, Common variable immunodeficiency, Allergy, Primary immunodeficiency, HLA, Antibiotic hypersensitivity

## Abstract

Hereditary angioedema (HAE) and common variable immunodeficiency (CVID) are rare disorders with heterogeneous clinical presentations that pose significant diagnostic challenges. HAE is characterized by recurrent episodes of subcutaneous or submucosal edema affecting the skin, gastrointestinal tract, or airway, whereas CVID presents with variable immune dysfunction, recurrent infections, and impaired vaccine responses. The clinical manifestations of these conditions can mimic allergic reactions, drug hypersensitivity, mast cell disorders, autoimmune diseases, and other immunodeficiencies, making careful differential diagnosis essential. Here, we present an exceptionally rare case of a patient with concurrent HAE type I and CVID, initially evaluated for suspected antibiotic hypersensitivity, highlighting the complexity of diagnosis and therapeutic decision-making. Laboratory findings confirmed both conditions, showing low C1 inhibitor levels and activity, markedly reduced serum concentrations of immunoglobulin G (including subclasses G1, G2, and G4), immunoglobulin A, and immunoglobulin M, along with a poor specific antibody response to vaccines. Skin allergy and oral provocation tests were negative, effectively ruling out antibiotic hypersensitivity. The genetic analysis identified a heterozygous pathogenic *SERPING1* variant, c.498C > A (p.Asn166Lys), previously reported in 1 Romanian and 1 Czech case, confirming the diagnosis of HAE. Human leukocyte antigen (HLA) genotyping revealed HLA-B∗35:01 and B∗44:01, with homozygosity for HLA-C∗04:01, alleles previously associated with increased susceptibility to CVID. Using this case as a framework, we review the literature on coexisting HAE and CVID, examining clinical presentations, diagnostic approaches, biomarker profiles, and reported HLA associations. The review highlights the critical importance of a comprehensive and personalized therapeutic approach, combining prophylactic and on-demand treatment for HAE with individualized immunoglobulin replacement for CVID. Integrating detailed clinical assessment with immunologic and genetic testing is essential for the management of rare, complex disorders, and further studies are needed to clarify potential immunogenetic links between HAE and CVID.

## Introduction

Hereditary angioedema (HAE) is a rare genetic disorder transmitted in an autosomal dominant pattern. It is characterized by recurrent episodes of non-pitting, self-limiting edema affecting the skin and/or submucosal tissues. The swelling can involve the face, trunk, extremities, gastrointestinal tract, genitals, and, most critically, the upper airways. Laryngeal and glottic edema are particularly dangerous, as they can lead to airway obstruction and become life-threatening.^1^

The prevalence of HAE varies by region and is estimated to range from 1:50,000 to 1:100,000, although the true global prevalence remains uncertain,^2^ with reported rates generally lower in Asia and Africa when compared to Europe and North America.^3^ In a German study conducted between 2016 and 2021, the prevalence among pediatric patients under 12 years of age was reported to range from 2.51:100,000 to 1.02:100,000.^4^

HAE can be caused by several genetic mutations such as:

• C1 inhibitor deficiency (Type 1) and C1 inhibitor dysfunction (Type 2) are due to mutations in the *SERPING1* gene, with over 700 variants identified until now,^1^ some of them occurring as de novo mutations. These genetic defects determine the protein synthesis defects, resulting in low concentrations of functional C1-INH;^5^
• HAE with normal C1 inhibitor is caused by mutations in other genes, including *F12* (encoding factor XII),^6^ genes involved in vascular endothelium functions (angiopoietin-1,^7^ myoferlin), *PLG* (plasminogen),^8^ *KNG1* (kininogen 1), *HS3ST6* (heparan sulfate-glucosamine 3-O-sulfotransferase 6)^1^ and also 3 mutations identified in the *CPN1* gene which encodes for the catalytic subunit of carboxypeptidase N (CPN). CPN is an enzyme that regulates biologically active peptides like complement anaphylatoxins and kinins, and its deficiency contributes to disease pathology.^9^

Common variable immunodeficiency (CVID), the most common primary immunodeficiency, also called acquired hypogammaglobulinemia, is characterized by clinical heterogeneity. This clinical syndrome is considered “variable” because of the multiple potential sites of immune dysregulation, including intrinsic T-cell defects, B-cell function abnormalities, and alterations in tumor necrosis factor (TNF) receptors.^10^ CVID is diagnosed when serum IgG levels are below 5 g/L in adults, accompanied by low IgA and/or IgM, and when other causes of immunodeficiency, such as medications, physiological immaturity, protein loss or malignancy have been excluded. Diagnosis is generally made in patients older than 4 years, with demonstration of poor vaccine responses due to defective antibody production against both protein and carbohydrate antigens.^11^

The estimated prevalence of CVID varies across studies, ranging from 1:20,000 to 1:50,000 life births,^10^ while other reports suggest an international prevalence of 1:10,000 to 1:100,000.^12^

The etiology of CVID remains largely unknown, and its genetic background is highly complex. The genes identified in cohorts of CVID patients reflect the critical role of class switch recombination, the complex mechanism of B cell antigen signaling, activation and migration, as well as the long-term survival, maturation and maintenance of antibody-secreting B cells in the plasma cell stage.^11^ One study suggested that mutations in genes involved in Toll-like and T-cell receptor signaling, which affect T-B cell interactions, may contribute to B-cell dysfunction.^13^

We present an exceptionally rare case of a patient with concurrent HAE and CVID, a coexistence that posed significant diagnostic and therapeutic challenges. Using this case as a reference, we review the current literature on each condition, discussing diagnostic strategies, management options, and outcomes. Our analysis integrates classical laboratory assessments, such as C1 inhibitor levels and activity, serum immunoglobulins, and vaccine-specific antibody responses, with evaluation of genetic predisposition, including HLA polymorphisms linked to CVID susceptibility and immune regulation. This integrated approach highlights the complexity of these overlapping disorders and underscores the importance of personalized diagnostic and therapeutic strategies.

## Case presentation

**Fig. 1:**
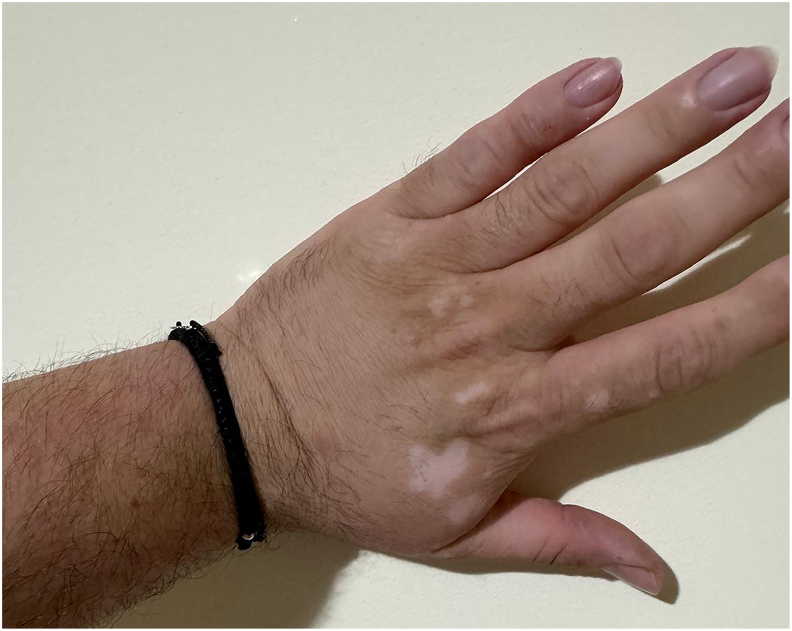
Depigmented lesions of vitiligo on the left hand

We report the case of a 34-year-old man with a family history of HAE type I involving multiple relatives (paternal grandmother, father, brother, and daughter), but no family history of CVID. His personal medical history is notable for recurrent infections, including respiratory infections with *Moraxella catarrhalis* and *Haemophilus influenzae* (since childhood: 8 episodes of rhinosinusitis and otitis per year, and 3 episodes of pneumonia per year), and gastrointestinal infections (7–8 episodes of enterocolitis and gastroenteritis per year). He has undergone surgical intervention (appendectomy in 2002) and has a history of dermatologic autoimmune diseases, including vitiligo (onset in 2014 on the hands, now also affecting the arms and genital region) and psoriasis (onset in 2012 on the elbows, currently treated with clobetasol propionate, Fig. 1).

The patient addressed to our clinic in May 2024, showing a persistent, irritating cough lasting over 1 month, following an acute upper respiratory tract infection treated with amoxicillin/clavulanic acid 875/125 mg twice daily for 10 days. A few days after completing the antibiotic course, he experienced a severe episode of cough accompanied by dysphonia and inspiratory dyspnea, followed by a brief loss of consciousness, which resulted in a minor car accident. The exact duration of these events could not be precisely determined by the patient. The neurological evaluation, cranio-cerebral CT scan, and electroencephalography performed in the emergency department, excluded a neurological cause. Cardiology assessment with electrocardiogram and echocardiogram ruled out cardiac causes. Otorhinolaryngology examination revealed laryngeal congestion without edema, bilateral maxillary and left sphenoidal sinusitis, and nasal septum deviation. Pulmonology evaluation diagnosed bronchial asthma and recommended inhaled budesonide/formoterol 160/4.5 μg, administered twice daily. A subsequent thoraco-abdominal CT scan revealed hypersensitivity pneumonitis, correlating with a 4-year occupational exposure to chemical substances in the auto paint industry, as well as an accessory spleen and hepatomegaly.

After 2 weeks, and respectively 1 month from the first episode, the patient experienced 2 additional episodes of cough-induced syncope. A gastroenterological evaluation diagnosed chronic gastritis and gastroesophageal reflux disease (GERD). Following a two-week course of pantoprazole 40 mg daily, and a histamine H_2_ receptor antagonist (famotidine 40 mg) daily, no further episodes of cough or syncope occurred, suggesting that the cough was related to GERD.

**Fig. 2:**
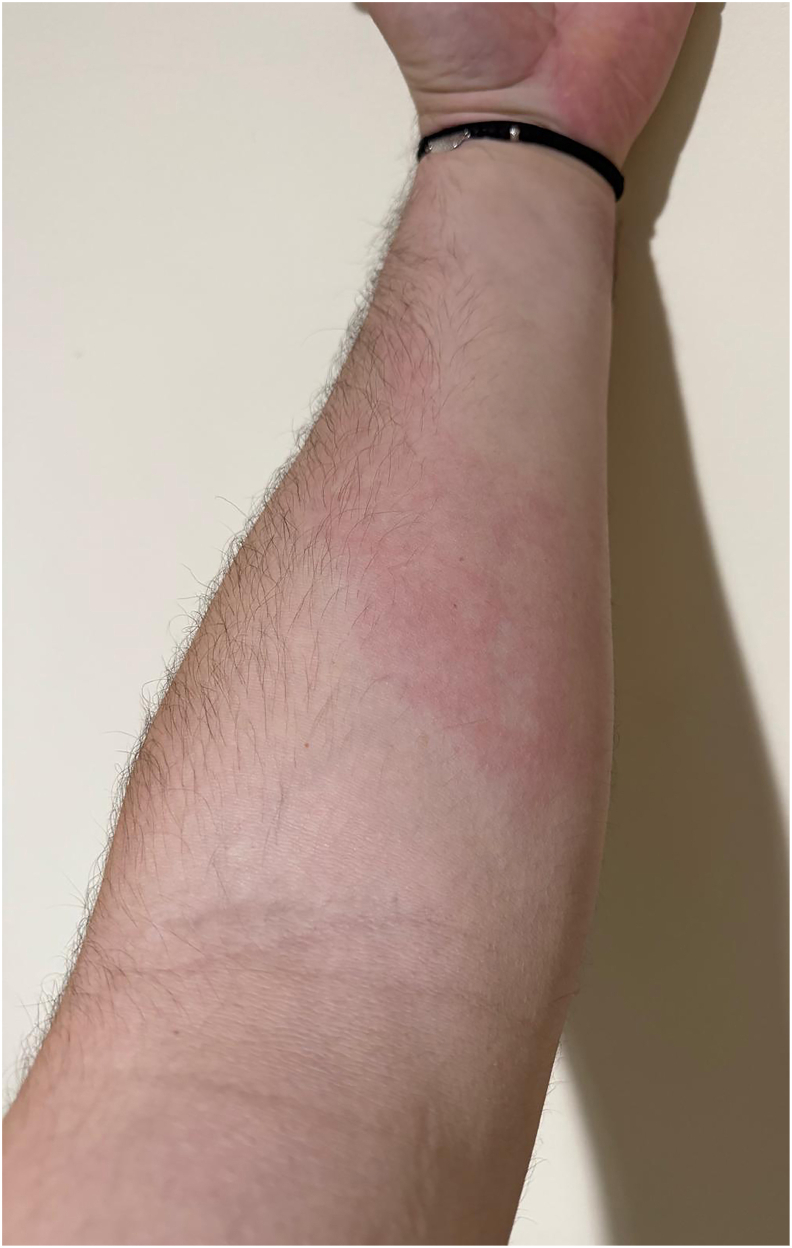
Hereditary angioedema (HAE) attack involving the right thigh.

**Fig. 3:**
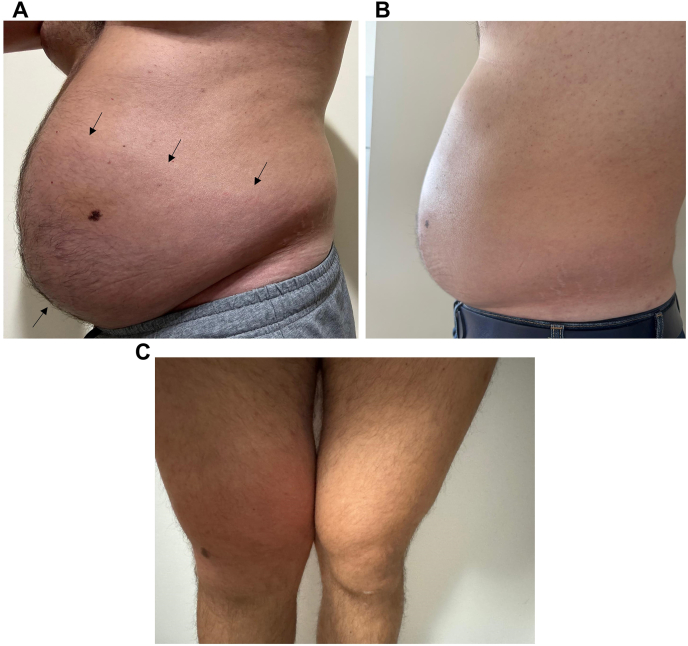
(A) Hereditary angioedema (HAE) attack involving the abdomen. Arrows mark the extent of visible angioedema. (B) Normal abdomen between HAE attack episodes. (C) HAE attack involving the right thigh

The patient also had a seven-year history of recurrent extremity swelling (without urticarial wheals, lasting 3–4 days, Fig. 2) and, since childhood, recurrent abdominal attacks occurring approximately twice a year (Fig. 3), characterized by abdominal pain, vomiting, diarrhea, and bloating lasting about 3 days, often triggered by infectious episodes, and without associated upper airway edema.

Blood analysis showed consistent low level and impaired function of C1 inhibitor over time (148 mg/L with 40% activity in 2024, and 103 mg/L with 39% activity in 2026), along with low levels of IgG (IgG1, IgG2, IgG4), IgA, IgM, and IgE, as well as decreased total hemolytic complement (CH100) and poor vaccine responses. Lymphocyte immunophenotyping revealed B cells (CD19^+^ lymphocytes) at the lower limit of normal range (Table 1). The patient had no history of angiotensin-converting enzyme inhibitor (ACEI) treatment or other medication that might cause hypogammaglobulinemia.

**Table 1:** Laboratory immunological evaluation.

Laboratory tests	Patient value	Normal value
C1 inhibitor protein level (between attacks)	↓ 148 mg/L (2024) ↓ 103 mg/L (2026)	210–390 mg/L
C1 inhibitor function (between attacks)	↓ 40% (2024) ↓ 39% (2026)	70–130%
C1q	184 mg/L	120–196 mg/L
C3	167.8 ± 5.08 mg/dl (between attacks: mean ± SEM; *n* = 9) 181 ± 4.5 mg/dl (1 day after attack onset: mean ± SEM; *n* = 2; no change: *p* = 0.2576)	82–185 mg/dl
C4	26.08 ± 0.79 mg/dl (between attacks: mean ± SEM; *n* = 9) 19.6 ± 0.4 mg/dl (1 day after attack onset: mean ± SEM; *n* = 2; relative decrease, *p* = 0.005)	15–53 mg/dl
IgG	↓ 378 mg/dl	540–1822 mg/dl
IgM	↓ 10 mg/dl	22–240 mg/dl
IgE	↓ <1 UI/ml	0–87 UI/ml
IgA	↓ <5 mg/dl	63–484 mg/dl
IgG1 subclass	↓ 2.72 g/L	4.05–10.11 g/L
IgG2 subclass	↓ 1.41 g/L	1.69–7.86 g/L
IgG4 subclass	↓ 0.0096 g/L	0.03–2.01 g/L
CH100	↓ <3 U/ml	30–75 U/ml
Pneumococcal titer (1 month post-vaccination)	↓ <0.3 mg/L	>10 mg/L
CD4^+^ T cells	699 cells/μL	332–1642 cells/μL
CD8^+^ T cells	538 cells/μL	170–811 cells/μL
B cells (CD19+)	160 cells/μL (7%)	78–899 cells/μL (7–23%)
NK cells (CD16+/CD56+)	736 cells/μL	67–1134 cells/μL

SEM, standard error of the mean; *n*= number of repeats.

Allergy testing, including prick test for aeroallergens and molecular allergy assays, was negative. The complete autoimmune panel (including anti-ribonucleoprotein complex [RNP], anti-Smith [Sm], anti-Ro RNA-associated proteins [SS-A], anti-La protein [SS-B], anti-topoisomerase I [Scl-70], anti-polymyositis/scleroderma [PM-Scl 100], anti-histidyl-tRNA synthetase [Jo-1], anti-Centromer B, anti-Proliferating Cell Nuclear Antigen [PCNA], anti-double-stranded DNA [dsDNA], anti-Nucleozom, anti-Histone, anti-ribosomal Protein P, antimitochondrial M2 [AMA-M2], anti-dense fine speckled protein [DFS70], anti-cytoplasmic antineutrophil cytoplasmic [cANCA], anti-perinuclear anti-neutrophil cytoplasmic [pANCA], anti-Liver Kidney Microsomal Type 1 [LKM], anti-transglutaminase, anti-Thyroid Peroxidase antibodies) and beta-2-microglobulin were within normal limits. Screening for infectious agents, including HIV, Epstein–Barr virus, Cytomegalovirus, Toxoplasmosis, hepatitis B and C viruses, Herpes simplex virus types 1 and 2, *Bordetella pertussis*, *Mycobacterium tuberculosis*, and *Helicobacter pylori*, was negative. No evidence of lymphoma, marked gammaglobulins loss, or malignancies was detected. To further exclude an acquired form of C1 inhibitor deficiency associated with autoimmune disorders, serum C1q levels were measured and found to be within the normal range. This result supports the diagnosis of HAE, as C1q levels are typically normal in HAE but reduced in acquired angioedema associated with autoimmune diseases such as systemic lupus erythematosus.^1^

In addition to the primary diagnoses of HAE and CVID, complement activation, inflammatory biomarkers, and human leukocyte antigen (HLA) genotype were thoroughly evaluated to better define the patient's immune status and genetic background.

To assess complement activation, serum C3 and C4 levels were measured. C3 levels remained within the normal range and did not show significant variation between attack and remission periods (181 ± 4.5 mg/dl vs. 167.8 ± 5.08 mg/dl, *p* = 0.2576). The anaphylatoxin C3a decreased from 97 ng/mL pre-treatment to 46 ng/mL post-treatment with immunoglobulin and C1 esterase inhibitor (human), remaining within the laboratory reference interval (15–200 ng/mL). This decline is consistent with reduction of complement activation following targeted therapy for C1-INH deficiency, which is known to modulate generation of complement fragments such as C3a/C4a/C5a during attacks of HAE.^14^, ^15^ Interestingly, although repeated C4 levels remained within the normal range during both attack and remission periods, they were significantly lower during attacks than during remission (19.6 ± 0.4 mg/dl vs. 26.08 ± 0.79 mg/dl, *p* = 0.005). This observation may be explained by the concomitant presence of CVID. Activation of the classical complement pathway depends largely on antigen–antibody complexes, particularly IgG and IgM. Reduced immunoglobulin levels in CVID may therefore lead to diminished activation of the classical complement pathway and less pronounced complement consumption, which could account for the normal C4 levels observed in this patient despite the presence of HAE. Conversely, MMP-8 (neutrophil collagenase) increased from 40 ng/mL to 57 ng/mL and remained markedly above the commonly cited normal range (<10 ng/mL). Elevated circulating MMP-8 is a non-specific marker of neutrophil-driven inflammation and tissue remodeling and has been reported as increased in diverse inflammatory and infectious conditions; although MMP-8 is not established as a disease-specific biomarker for HAE or CVID, its persistent elevation suggests ongoing inflammatory/remodeling activity that is not fully suppressed by the administered therapies.^16^

Genetic analysis of an extended HAE gene panel (*SERPING1*, *ANGPT1* exon 2, *F12* exon 9, *KNG1* exon 10, and *PLG* exon 9)^1^ was performed in an external private laboratory using next-generation sequencing. Target regions, including coding exons, flanking intronic sequences, and additional disease-relevant non-coding regions, were enriched by in-solution hybridization and sequenced on an Illumina NovaSeq 6000 platform. The analysis identified a heterozygous *SERPING1* variant, c.498C > A (p.Asn166Lys), classified as pathogenic in 2 previously reported cases, one of Romanian origin^17^ and another from the Czech Republic,^18^ thereby confirming the diagnosis of HAE.

HLA six-locus NGS genotyping in our laboratory using GenDx IVD kits (NGSgo®-MX6-1 and NGSgo® Library Full Kit)^19^ on the Illumina iSeq 100 platform revealed several particularities, including for HLA-B, which showed the genotype HLA-B∗35:03:01:01–B∗44:03:01:01 (Table 2). The combined HLA-B∗35–B∗44 haplotype has an estimated frequency of 2.16% in the Romanian population but is reported more frequently among individuals with autoimmune diseases such as myasthenia gravis (5%)^20^ and HLA-B27–negative spondyloarthritis (3%).^19^ Importantly, the HLA haplotype B44-DR7 has been associated with CVID,^21^ which is particularly relevant in this patient who carries both B∗44 and DRB1∗07 alleles. Although this haplotype is not directly causal for HAE or CVID, it may indicate a genetic background favoring immune dysregulation and altered tolerance, consistent with the patient's clinical profile. Furthermore, the patient is homozygous for HLA-C∗04:01. In a recent study of CVID patients from Turkey, the frequencies of both HLA-B∗35 and HLA-C∗04 were significantly higher in patients compared to healthy controls, supporting a potential role for these alleles in disease susceptibility.^22^ While HLA-C∗04:01 allele is not directly implicated in HAE, its homozygous presence may contribute to broader immune imbalance, consistent with the overlap phenotype of CVID with immune dysregulation and persistent inflammation. Interestingly, our CVID patient exhibited a normal B-cell count, in line with previous reports.^23^ In that study, CVID patients carrying HLA-B∗44 or HLA-B∗08 showed higher circulating B-cell numbers compared to non-carriers, suggesting that the mechanisms underlying immunodeficiency may vary between different HLA-defined subgroups.

**Table 2:** Results of the HLA class I and class II NGS genotyping.

	Allele 1	Allele 2
HLA-A	02:01:01:01	23:01:01:01
HLA-B	35:03:01:01	44:03:01:01
HLA-C	04:01:01:01	04:01:01:01
HLA-DRB1	07:01:01:01	13:02:01:02
HLA-DQB1	02:02:01:01	06:03:01:01
HLA-DPB1	04:01:01:03	04:01:01:10

We also performed skin prick and intradermal tests, followed by oral provocation tests for amoxicillin and clavulanic acid, due to the temporal association with symptom onset; all tests were negative.

The patient is currently receiving treatment for HAE type I with intravenous plasma-derived C1 inhibitor (C1-INH) for short-term preprocedural prophylaxis, and subcutaneous Icatibant for acute attacks (initiated in May 2024), with a stable course of angioedema episodes. For CVID, he is treated with subcutaneous facilitated human immunoglobulin (since October 2024), resulting in a significant improvement in his infection pattern, from 8 upper respiratory tract infections per year to only 2 to 3, with no further episodes of pneumonia or gastrointestinal infections.

## Hereditary angioedema: Clinical challenges and management approaches

HAE attacks can occur either spontaneously or be triggered by stress and/or trauma,^24^ infections, angiotensin-converting enzyme inhibitors (ACEI) treatment, hormonal contraceptives, pregnancy, and menstruation, due in part to the effect of estrogen in reducing C1 inhibitor levels through mechanisms that are not yet fully elucidated,^25^ as well as by other unidentified factors. In our case many of the triggers were infections, stress and trauma, but also unidentified factors.

Several studies emphasize the importance of awareness and prevention of comorbid conditions in patients with HAE. These patients may develop comorbidities such as cardiovascular diseases, including arterial and venous thromboembolic events,^26^ hypertension, cerebral infarction, ischemic heart disease, pulmonary embolism, and hyperlipidemia, as well as autoimmune diseases such as systemic lupus erythematosus, autoimmune thyroiditis,^27^ glomerulonephritis, and Sjögren's syndrome.^28^ In our patient, the associated autoimmune diseases were vitiligo and psoriasis.

The diagnosis of HAE is based on clinical manifestations and laboratory findings. Upper airway edema is among the most critical symptoms, as it can be a life-threatening condition.^29^ The hallmark of HAE is recurrent angioedema attacks, which may involve the face, trunk, extremities, gastrointestinal tract, genitalia, or airways. These attack episodes are not associated with urticaria or pruritus, but may be preceded by prodromal symptoms such as erythema marginatum, a nonpruritic macular rash,^30^ fatigue, malaise, or musculoskeletal pain. The angioedema typically lasts 3–5 days and does not respond to antihistamines, omalizumab, corticosteroids, or epinephrine. The clinical presentation can be misattributed to appendicitis or gastroenterocolitis, due to symptoms such as abdominal pain, vomiting, diarrhea and nausea.^31^ In our patient, given the lack of confirmed gastrointestinal infections, those episodes of enterocolitis and gastroenteritis could possibly have been misdiagnosed manifestations of HAE.

The laboratory tests in HAE type I typically reveal low C1 inhibitor protein levels and function. In HAE type II, C1 inhibitor protein levels are normal or elevated, but function is reduced.^1^ HAE with normal C1-INH (formerly known as HAE type III) is associated with diverse genetic variants and normal baseline C1-INH levels and function, although transient decreases in C1-INH activity may occur during attacks in some patients. Additionally, C4 levels are recommended to be measured, particularly during the acute attacks, when they are often low;^32^ between attacks, C4 may be normal, with a reported sensitivity of 80%. In our patients, C4 levels were normal between attacks, as shown in Table 1, but decreased by approximately 20% during attacks, although still remaining within the normal range. This is indeed a rare condition; however, it has been previously described in another patient from the United Kingdom.^33^ Additionally, in our patient, the persistently normal C4 levels may be further explained by the concomitant presence of CVID, which might lead to reduced consumption of C4, since classical complement pathway activation is largely dependent on IgG and IgM levels. By contrast, data regarding overall complement activation remain heterogeneous; certain studies describe increased complement split products or altered complement function in subsets of patients, whereas others report near-normal levels. Therefore, reduction of C3a serum levels in our patient after therapy, should be interpreted as improvement in complement regulation rather than complete restoration of immune homeostasis. Other laboratory markers include D-dimer, which may rise during acute HAE attacks, and C1 and/or C3 levels, which were normal in our patients. Next-generation sequencing testing confirmed the diagnosis of HAE, revealing the presence of a rare heterozygous *SERPING1* variant, c.498C > A (p.Asn166Lys), classified as pathogenic in another 2 patients, one of Romanian origin^17^ and another from the Czech Republic.^18^

The treatment for this disease is particularized for solving the acute attacks and includes plasma-derived C1-INH,^34^ recombinant human C1-INH,^35^ bradykinin receptor antagonist like Icatibant,^36^ plasma kallikrein inhibitor like Ecallantide,^37^ and when these are not available fresh frozen plasma (FFP).^38^ For the short-term preprocedural prevention, procedures such as facial surgery, dental surgery, tooth extraction, tonsillectomy, bronchoscopy, endotracheal intubation, esophagogastroduodenoscopy require treatment with plasma-derived C1-INH or recombinant human C1-INH.^38^ For long-term prophylaxis, options include plasma kallikrein inhibitors such as Berotralstat (an oral therapy) and monoclonal antibodies such as Lanadelumab (a subcutaneous therapy).^39^ Other agents include attenuated androgens like Danazol and Oxandrolone, which are associated with numerous side effects, as well as Tranexamic Acid.^1^ New therapies under investigation for the long-term prophylactic treatment include Donidalorsen, an inhibitor of hepatic prekallikrein production,^40^ and Garadacimab, a fully human anti-activated Factor XII monoclonal antibody.^41^ Our patient receives intravenous plasma-derived C1 inhibitor for the short-term preprocedural prevention, and subcutaneous Icatibant for acute attacks, with similar frequency and course of angioedema episodes, and without any life-threatening events.

## Common variable immunodeficiency: clinical challenges and management approaches

The diagnosis of CVID is based on exclusion criteria. In recent years, several organizations and research groups have attempted to harmonize the diagnostic criteria. Thus, CVID may be considered in a patient with marked decrease in serum IgG levels, with a proposed lower limit of 5 g/L in adults (at least 2 standard deviations below the mean for age) and a significant reduction at least in 1 of the other isotypes IgM or IgA. All of the following cirteria should also be met: onset of symptoms after the age of 2 years, absent isohemagglutinins and/or poor antibody response to vaccines (both T-dependent antigens, such as diphtheria, tetanus toxoid, and *Haemophilus influenzae* type B, and T-independent antigens, such as the pneumococcal polysaccharide vaccine) and exclusion of other causes of hypogammaglobulinemia. These include drug induced causes (eg, glucocorticosteroids, antiepileptic drugs, rituximab), genetic or chromosomal defects, infectious diseases (HIV, congenital infections with rubella virus, cytomegalovirus, *Toxoplasma gondii*, or EBV), malignancies (such as chronic lymphocytic leukemia or lymphomas) and conditions causing excessive immunoglobulin loss (eg, severe burns, nephrosis, protein-losing enteropathy, or lymphangiectasia).^42^

In our case, all diagnostic criteria were fulfilled. Additional tests that can be performed include complement assays (investigating the serum levels of CH50, C3, C4), quantitative IgG subclass measurement, quantification of blood T- and B- cell subpopulations, using specific surface biomarkers of T cells (CD3, CD4, CD8, TCRαβ, TCRγδ), and B cells (CD19; CD20; CD21; Ig μ, δ, γ, α, κ, λ; Ig-associated molecules). Further investigation may involve HLA genotyping, evaluation of CD40, CD40 ligand expressions, and genetic testing. The clinical presentation of patients with CVID typically include recurrent upper respiratory tract infections such as otitis media and sinusitis caused by encapsulated bacteria (*Haemophilus influenzae*, *Streptococcus pneumoniae*), as well as pulmonary infections due to a broader range of pathogens such as, including *Pseudomonas aeruginosa* and *Staphylococcus aureus*. Some CVID patients develop chronic diarrhea, most commonly due to *Giardia lamblia,* and show an increased susceptibility to infections with *Salmonella, Shigella* and *Campylobacter*. In addition, they are at increased risk for gastrointestinal and lymphoid malignancies,^43^ unusual recurrent urinary tract or uterine/cervical infections, arthritis, chronic lung disease such as bronchiectasis or obstructive/restrictive disorders, mediastinal lymphadenopathy, sarcoidosis, diffuse granulomatous disease, and various autoimmunity conditions (immune thrombocytopenic purpura, pernicious anemia, autoimmune hemolytic anemia, autoimmune neutropenia, inflammatory bowel disease, seronegative arthritis, vasculitis, Sjögren syndrome, uveitis, systemic lupus erythematosus, thyroiditis, alopecia, vitiligo, hepatitis, primary biliary cirrhosis).^44^ Other associated findings include splenomegaly, hepatomegaly, osteoporosis,^42^ cellulitis, impetigo, measles, warts and eczema.^44^ Patients with CVID may rarely present with associated syndromes such as Pitt–Hopkins syndrome^45^ and amyloidosis.^46^ In our case, chronic obstructive disease (represented by bronchial asthma), vitiligo, psoriasis and hepatomegaly were the conditions associated with CVID. Some studies classify CVID in 2 phenotypes: one characterized mainly by recurrent infections, and another (“CVID+”) in which autoimmune and inflammatory complications are the predominant manifestations.^47^

The standard treatment for CVID is immunoglobulin replacement therapy with IgG, which can be administered intravenously, subcutaneously, or subcutaneously with hyaluronidase facilitation. Administration is typically performed at 3- or 4-week intervals, depending on the patient's clinical response and individual needs. The majority of national and international guidelines recommend a dosage of 0.4–0.5 g/kg/month for intravenous immunoglobulin (IVG) and 0.4–0.6 g/kg/month for subcutaneous immunoglobulin (SCIG). In patients with splenomegaly or enteropathy, higher doses, ranging from 0.6 to 0.8 g/kg/month, may be indicated. Our patient is currently receiving 0.4 g/kg/month hyaluronidase-facilitated SCIG with good clinical response and a marked reduction in infection frequency. Other treatment methods described in literature include: antibiotics for recurrent infections and bronchiectasis, corticosteroids for granulomatous disease, interstitial lung disease, lymphoproliferation and autoimmune cytopenia, as well as rituximab and splenectomy for autoimmune hemolytic anemia and immune thrombocytopenic purpura. For CVID-associated enteropathy, therapies such as 5-aminosalicylic acid and/or budesonide, azathioprine, 6-mercaptopurine, or infliximab have been used. Anti-TNF antibody therapy has shown benefits in some cases of interstitial lung disease. Treatment protocols for lymphoma in CVID patients generally follow the same principles as those used in immunocompetent individuals. Stem cell therapy remains experimental with limited reported experience,^42^ while single or multiorgan transplantation may be considered for patients with end-stage organ involvement unresponsive to medical therapy.^48^

## Coexistence of 2 rare diseases: Exploring genetic susceptibility

To investigate a potential genetic susceptibility underlying the coexistence of these 2 rare diseases, we performed HLA NGS genotyping across 6 loci. Only a few studies have explored the potential link between HAE and HLA. In 1 study, no significant association between HLA alleles and HAE was observed.^49^ In another study, given that genes for certain complement components (C2, C4, and C8) are located near HLA genes on chromosome 6, researchers examined the possible linkage of the HAE gene locus to HLA in 2 families. The evidence did not support a close linkage between HLA and HAE.^50^^,^^51^

By contrast, the association between CVID and HLA has been more extensively investigated. In 1 study, immunophenotyping, including assessment of HLA-DR expression on T-lymphocyte subsets, demonstrated that CD8^+^DR^+^ T cells exhibited a cytotoxic phenotype in a subset of CVID patients.^52^ Another study reported that CVID patients had higher levels of CD8^+^DR^+^T cells compared to controls.^53^

In another study, individuals carrying haplotype 1 (HLA-DQB1 0201, HLA-DR3, C4B-Sf, C4A-0, G11-15, Bf-0.4, C2-a, HSP-7.5, TNF-5, HLA-B8, HLA-A1) and haplotype 2 (HLA-DQB1 0201, HLA-DR7, C4B-S, C4A-L, G11-4.5, Bf-0.6, C2-b, HSP-9, TNF-9, HLA-B44, HLA-A29) were shown to have an increased risk of developing CVID.^54^ Our patient carries the haplotype HLA-B∗44 and DRB1∗07 (related to haplotype2), which is associated with an increased genetic predisposition to developing CVID. Additionally, in large subsets of CVID patients, there is an increased degree of conservation within the HLA class III region, particularly in the subregion between the C4B and C2 genes. Animal studies have shown that reduced immune responses to T-dependent and independent antigens are related to the deficiencies of the classical pathway complement components, including C4. In these complement deficient animals, both the primary and secondary immune responses are decreased, and isotype switching during secondary responses is defective. The mechanisms by which complement participate in antibody responses involve signal transduction through the B cell surface receptors, CR1 and CR2, which recognize activation fragments of C4 and C3. Additionally, defective isotype switching is supported by the observation that human with similar complement deficiencies show decreased serum concentration of IgG4.^55^ This could explain why our patient presents with both rare diseases.

Our patient also carries HLA-B∗35 and is homozygous for HLA-C∗04. In a Turkish population, both HLA-B∗35 and C∗04 alleles were found to be more frequent in the CVID group compared to healthy controls.^22^ In conclusion, research has identified at least 3 susceptibility loci on the short arm of chromosome 6 within the major histocompatibility complex: 1 within the class II region (HLA-DRB1 locus) and 2 within the class I region (HLA-B and HLA-C loci), all potentially contributing to an increased risk of developing immunodeficiency disorders.^43^

To date, only 1 similar case has previously been described in the literature.^56^ The report described a 48-year-old patient presenting with recurrent angioedema who was suspected to have both HAE and CVID. In this case, mutations in the *SERPING1* gene or other genes associated with HAE were not demonstrated, and while IgG levels were reduced (4.4–4.6 g/L), IgA and IgM levels, which are required for a definite diagnosis of CVID,^42^ were not assessed, preventing definitive confirmation of the combined diagnosis. Interestingly, similar to our patient, the individual exhibited reduced C1-INH functional activity (39–47%), with complement components C3, C4, and C1q remaining within the normal range. Management included C1-esterase inhibitor replacement therapy for angioedema attacks and subcutaneous immunoglobulin therapy to address the underlying antibody deficiency.

## Conclusions

We report an uncommon case involving the coexistence of 2 rare diseases in the same patient—HAE type I and CVID. To date, only 1 previous patient suspected of having both HAE and CVID has been described,^56^ while another report noted HAE in a patient whose father had CVID.^57^ Importantly, our presented case involved thorough laboratory and clinical investigations and represents the first report of a confirmed dual diagnosis in a single individual. This case highlights the significant diagnostic and therapeutic challenges posed by the simultaneous presence of these disorders. Using this patient as a model, we reviewed the literature on coexisting HAE and CVID, discussing clinical presentations, laboratory and immunogenetic biomarkers, differential diagnoses, and therapeutic strategies. The case emphasizes the importance of a comprehensive and personalized approach, integrating prophylactic and on-demand HAE therapy with tailored immunoglobulin replacement for CVID. Although immune dysfunction has been studied separately in patients with CVID or HAE, further research is required to clarify potential shared pathogenic mechanisms, including the role of genetic susceptibility and HLA polymorphisms. This combined case report and literature review provides a framework for understanding the complex interplay between these rare disorders and may guide future diagnostic and therapeutic strategies.

## Abbreviations

HAE: Hereditary angioedema; CVID: Common variable immunodeficiency; C1-INH: C1 inhibitor; CPN: Carboxypeptidase N; Ig: Immunoglobulin; CT: Computer tomography; GERD: Gastroesophageal reflux disease; CH100: Total hemolytic complement; ACEI: Angiotensin-converting enzyme inhibitor; HIV: Human immunodeficiency virus; NK: Natural killer; HLA: Human leukocyte antigen; C: Complement; DBS: Dried blood spot; PLAUR: Plasminogen-activator-urokinase-receptor; u- PAR: urokinase- type plasminogen activator; AKT2: Serine/Threonine Kinase 2; FFP: Fresh frozen plasma; CR: Complement receptor; EBV: Epstein–Barr virus; Anti-TNF: Anti-tumor necrosis factor.

## Data availability statement

All data supporting the findings of this study are fully presented within the article.

## Author contributions

DU evaluated the patient upon admission, managed aftercare, reported the case, drafted and edited sections of the manuscript, edited figures, provided clinical supervision, and offered therapeutic guidance. DVM and REI collected relevant background information. DU, DVM, and MPT prepared the initial draft of the manuscript. MPT conducted part of the immunologic evaluations, critically revised the manuscript, performed the final revision before submission, and secured the funding. All authors contributed to the work and approved the submitted version.

## Ethics statement

The study was conducted in accordance with the Declaration of Helsinki and approved by the Ethics Committees of the *St. Spiridon* Clinical County Hospital of Iasi and *Grigore T. Popa* University of Medicine and Pharmacy Iasi (IRB number: 629/20.07.2025). The patient gave written informed consent for participation in this case report and to publish any accompanying images.

## Authors’ consent to publish statement

The patient gave written informed consent for publication of this case report and any accompanying images. A copy of the written consent is available for review by the Editor-in-Chief of this journal.

## Declaration of generative AI and AI-assisted technologies

Nothing to disclose.

## Funding

This study was funded by a project under The Health Program (PS) 2021–2027, Policy Objective 1, Priority 5, project title “Development of translational research for vaccines, serums and other biological drugs—Acronym CANTAVAC 2.0”, SMIS code 326920.

## Declaration of competing interests

The authors declare that they have no competing interests.
